# Effect of floor type on the performance, physiological and behavioural responses of finishing beef steers

**DOI:** 10.1186/s13028-015-0162-7

**Published:** 2015-10-31

**Authors:** Bernadette Earley, Barry McDonnell, Edward G. O’Riordan

**Affiliations:** Animal and Bioscience Research Department, Animal and Grassland Research and Innovation Centre, Teagasc, Grange, Dunsany, Co. Meath Ireland; Livestock Systems Research Department, Teagasc, Animal and Grassland Research and Innovation Centre, Grange, Dunsany, Co. Meath Ireland; College of Agriculture, Food Science and Veterinary Medicine, University College Dublin, Belfield, Dublin 4, Ireland

**Keywords:** Housing, Animal welfare, Concrete slat, Rubber mat

## Abstract

**Background:**

The study objective was to investigate the effect of bare concrete slats (Control), two types of mats [(Easyfix mats (mat 1) and Irish Custom Extruder mats (mat 2)] fitted on top of concrete slats, and wood-chip to simulate deep bedding (wood-chip placed on top of a plastic membrane overlying the concrete slats) on performance, physiological and behavioral responses of finishing beef steers. One-hundred and forty-four finishing steers (503 kg; standard deviation 51.8 kg) were randomly assigned according to their breed (124 Continental cross and 20 Holstein–Friesian) and body weight to one of four treatments for 148 days. All steers were subjected to the same weighing, blood sampling (jugular venipuncture), dirt and hoof scoring pre study (day 0) and on days 23, 45, 65, 86, 107, 128 and 148 of the study. Cameras were fitted over each pen for 72 h recording over five periods and subsequent 10 min sampling scans were analysed.

**Results:**

Live weight gain and carcass characteristics were similar among treatments. The number of lesions on the hooves of the animals was greater (*P* < 0.05) on mats 1 and 2 and wood-chip treatments compared with the animals on the slats. Dirt scores were similar for the mat and slat treatments while the wood-chip treatment had greater dirt scores. Animals housed on either slats or wood-chip had similar lying times. The percent of animals lying was greater for animals housed on mat 1 and mat 2 compared with those housed on concrete slats and wood chips. Physiological variables showed no significant difference among treatments.

**Conclusions:**

In this exploratory study, the performance or welfare of steers was not adversely affected by slats, differing mat types or wood-chip as underfoot material.

## Background

A number of studies have been conducted to establish if animal welfare and performance during housing is affected by conditions underfoot [1–3]. The conventional housing system in most European countries is a group pen with fully slatted concrete floors. More recently, studies have focused on the potential benefits of providing cattle with cushioned flooring [4–6]. Cozzi et al. [7] reported that floor type significantly affected average daily gain in a study carried out on finishing bulls housed on fully slatted concrete floors, a perforated floor and a perforated floor coated with a rubber mattress. Mayer et al. [8] and Ruis-Heutinck et al. [9] have all noted significant alterations in the lying behaviour of bulls housed on a concrete floor, such as a higher proportion of atypical lying down and standing up movements and fewer periods lying when compared with bulls kept in pens with a bedded lying area. Lameness in cattle housed on concrete floors is a major concern. Modern tie-stalls having rubber mats and slats at the rear, have been shown to reduce lameness problems considerably [10]. In a veterinary context, the term stress in farm animals is characterized by abnormal or extreme adjustment in the behaviour and physiology of the animal to cope with adverse changes in its environment and management [11]. Increased knowledge of the repertoire of emotions experienced by animals has been compiled via the examination of the relationship between the threat evaluation process and the behavioural and physiological responses in the animal [12–14]. Research addressing animal welfare is largely focused on measurements of animal behaviour [15, 16] stress physiology [17], veterinary epidemiology, environmental physiology, environmental design, comparative psychology and studies of the behaviour of animal handlers, together with conventional fields such as nutrition and microbiology [11, 14, 18–22]. The hypothesis of the present study was that providing finishing steers with underfoot comfort would enhance animal performance and welfare variables. Therefore a study was designed to evaluate concrete slatted flooring with and without rubber mats, and wood-chips placed on concrete slats, on performance, dirtiness, hoof health and blood biological variables of steers, housed for a winter finishing duration of 148 days.

## Methods

### Ethical considerations

The study was planned and performed in a manner aiming to prevent any unnecessary pain and discomfort to the animals. All animal procedures performed in this study were conducted under experimental licence (B100/2869) from the Irish Department of Health and Children in accordance with the Cruelty to Animals Act 1876 and the European Communities (Amendment of Cruelty to Animals Act 1876) Regulation 2002 and 2005.

### Animals, experimental design and diet

The study was conducted at the Teagasc Beef Research Centre, Grange, Co. Meath in winter 2007. One-hundred and forty-four finishing steers were randomly assigned according to their breed (124 Continental cross and 20 Holstein–Friesian) and average body weight (503 kg; standard deviation 51.8 kg) to one of four treatments for 148 days. Each treatment contained 4 randomly assigned pens of 9 animals stocked at a density of 2.73 m^2^/head. All the experimental pens were located in a naturally ventilated building with a feed alley in the center and pens on both sides of the alley. The experimental pens were randomly located throughout the building. Animals were fed a total mixed ration (TMR) of silage and rolled barley on a 50:50 DM basis using an Abbey Vertical Mixer (VF 12) fitted with a Digi-star weighing system (Digi-Star Europe, Panningen, The Netherlands). The weighing system was calibrated at the beginning of every 2nd week before the animals were fed. The TMR was delivered once in the morning. Feed was weighed into each pen every day throughout the experiment and refusals were measured at pen level twice weekly. Animals had ad libitum access to drinking water. The TMR mix offered was sampled and submitted for chemical analysis [dry matter (DM), crude protein, ash and nitrogen, acid detergent fibre (ADF), pH and dry matter digestibility (DMD)]. Sample processing and chemical analysis for the silage and concentrates was as described by Tilley and Terry [23] and Owens et al. [24].

### Floor treatments

The dimension of the individual pens were 4.1 m × 6.0 m (24.6 m^2^) and the concrete slats (5 gang/pen) were supplied by Banagher Concrete Ltd., Offaly, Ireland. Each gang had the following dimensions; void length 945 mm × 3; void space 35 mm; slat length 3.5 m; slat rib width 170 mm; slat width 1.18 m.

EasyFix (mat 1; EasyFix^®^ Rubber Products Ltd. Ballinasloe, Co. Galway, Ireland) and Irish Custom Extruders (mat 2; Irish Custom Extruders Ltd., Finglas, Dublin, Ireland) mats were placed on top of the concrete slats for the respective treatment pens by commercial technicians. The entire pen was covered with the respective mat. The mats were slatted to match the concrete slats and each mat allowed drainage. Wood-chip served as the positive control. In the wood-chip bedded pens, a permeable plastic sheet was fitted underneath the wood-chip to prevent the wood-chip from falling through the concrete slats. On top of this plastic layer, 1000 kg of wood-chip was used to bed the animals to a depth of approximately 45 cm. Faecal matter was removed once a week from the wood-chip pens and the wood-chip bed was replenished. The bare concrete slats and mat treatments were never cleaned as the stocking density and void material allowed the faecal material and urine to pass through the void space. The animals remained inside throughout the study except for the periods when they were moved to a holding pen for blood sampling and weighing. The mean daily air temperature (°C) in the shed facility and environmental temperature were continuously recorded using Testo 175 data loggers (Radionics, Dublin, Ireland).

### Average daily live weight and carcass characteristics

Animals were weighed on day-1 and again on day 0 before assignment to treatment, and on days 23, 45, 65, 86, 107, 128 and 148 throughout the experimental period, and average daily live weight gain determined. Animals were slaughtered over a 2 day period (day 149 and day 150) using equal numbers from each of the experimental treatments. At slaughter carcass weights, dressing percentage, and kidney and channel fat weights were determined for each animal. Carcasses were graded mechanically for conformation and fat scores according to the EU beef carcass classification scheme (EC, 2006). Carcass weight gain was estimated by subtracting initial carcass weight, calculated by assuming an initial dressing percentage of 0.52, from final carcass weight. Kidney and channel fat was recorded for each animal.

### Behaviour

Behavioural observations were recorded over five 3-day periods (1–5) (Period 1, days 2, 3, 4; Period 2, days 25, 28, 29; Period 3, days 42, 43, 44; Period 4, days 56, 57, 58 and Period 5, days 129, 130 and 133). Animals were identified by their natural body markings. During the period of darkness the shed housing the animals was dimly lit using infra-red lights to allow visualizing of the animals on the CCTV Eneo cameras (Lynx, Dunsany, Co. Meath, Ireland). An individual Eneo camera was placed over each pen and the CCTV data were recorded. The cameras were connected to a video tape recorder (Panasonic AG6040) via a multi-vision system (Panasonic WJ-FS109, monochrome duplex multiplexer, Lynx, Dunsany, Co. Meath, Ireland) which allowed pictures from all cameras to be viewed on one screen at a time. The pictures from all the cameras were marked with individual pen number, time and date settings. The behavioural analysis was performed by a trained staff member. Steers were observed by instantaneous scan sampling of the CCTV recordings. The interval between scans was 10 min. Each steer was observed for behavioural activity and body contact. Counts of lying, standing, eating and drinking, mounting, head-butting, licking and grooming were recorded. In the behavioural activity category, steers were observed for the following: lying down: head supported by the neck, head not supported by the neck (chin on the floor, on the body or on another steer); standing: with or without moving; eating (head in the trough); drinking; head to head contact except while eating; head contact with the body of another steer; and mounting. In the contact category the criteria were: no body contact with other steers and contact with one, two or three steers; grooming refers to self grooming and allogrooming constitutes licking another animal.

### Dirt scores

All animals were scored for dirtiness at the start of the study (day 0) and on days 23, 45, 65, 86, 107, 128 and 148 of the study in accordance with the dirt scoring system of Scott and Kelly [25]. The dirt scoring was performed by a trained staff member. The left side of each animal was diagrammatically divided into 16 segments and each body segment was assigned a score between 1 (very clean) and 5 (very dirty). All scores were combined to obtain a total score for each animal, between 1 and 80, which equated to the sum of the scores for each of the 16 body segments.

### Hoof score condition

The general condition of the four hooves of each animal was recorded prior to (day 0) and at the end of the study (i.e. on retrieval of the hooves post-slaughter of the animals) by a trained staff member. A single observer who was experienced in hoof examination scored all the animals on both occasions and preliminary scoring was carried out to verify repeatability of results. The hooves were trimmed to allow visualization, and both claws per each of the four hooves were examined for the presence of lesions using the method of Greenough and Vermunt [26]. The dorsal area was examined for equal and unequal size and the plantar area for heel erosion, under run sole, digital dermatitis, inter-digital dermatitis and white line damage. The total number of lesions was averaged over all four hooves and the number each animal had obtained during the study was determined. Animals were observed for any signs of lameness every 3 weeks to coincide with animal weighings.

### Health

The general health status of the steers was recorded by a trained staff member. A complete record of any clinical symptoms by infection or injury and their veterinary treatment was maintained. All animals remained clinically healthy throughout the study period.

### Blood samples and assay procedures for physiological variables

Maintaining aseptic procedures, animals were blood sampled via jugular venipuncture on days 0, 23, 45, 65, 107, 128 and 148. For this procedure, the animals were moved to a holding pen with a squeeze chute facility and were blood sampled with minimal restraint. Blood sampling was carried out by the same experienced operator on each occasion and the time taken to collect the blood samples was less than 60 s/animal. Blood samples were collected into (1 × 6 mL) K_3_ethylenediaminetetraacetic acid (K_3_EDTA) tube (Vacuette, Cruinn Diagnostics, Ireland) for haematological analysis. Heparinized blood samples (1 × 9 mL) were collected and the plasma was separated by centrifugation 1600×*g* at 8 °C for 15 min (except for interferon (IFN)-γ at 300×*g*; at 8 °C for 15 min) and subsequently stored at −20 °C until assayed for analysis of;

#### Cortisol

Cortisol A competitive immunoassay (Correlate-EIA Cortisol, Assay Designs, Ann Harbor, MI, USA, catalogue number 901-701) was used for the quantitative determination of cortisol in plasma.

#### Total protein

The concentration of total protein in plasma was determined on an automatic clinical analyser (Olympus AU400 Clinical Analyser, Tokyo, Japan) using the reagents supplied by Olympus (catalogue number OSR6132) (Olympus UK Ltd., Voice house, Watford, Hertfordshire, WD24 4JL, UK). The composition of the reagents (at final concentration) required for this test were; sodium hydroxide (200 mmol/L), potassium sodium tartrate (32 mmol/L), copper sulphate (18.8 mmol/L) and potassium iodide (30 mmol/L), and following gently inversion the reagents were ready to use directly from the kit.

#### Albumin

The concentration of albumin was determined on an automatic clinical analyser (Olympus AU400 Clinical Analyser, Tokyo, Japan) using the reagents supplied by Olympus (catalogue number OSR6102). The composition of the reagents (at final concentration) required for this test were; succinate buffer (pH 4.2) (100 mmol/L) and bromocresol green (0.2 mmol/L), and following gently inversion the reagents were ready to use directly from the kit.

#### Creatine kinase

The activity of creatine kinase was determined on an automatic clinical analyser (Olympus AU400 Clinical Analyser, Tokyo, Japan) using the reagents supplied by Olympus (catalogue number OSR6179). The composition of the reagents (at final concentrations of reactive ingredients) required for this test were; imidazole (pH 6.5) (100 mmol/L), NADP (2 mmol/L), ADP (2 mmol/L), AMP (5 mmol/L), EDTA (2 mmol/L), glucose (20 mmol/L), creatine phosphate (30 mmol/L), *n*-acetlycysteine (0.2 mmol/L), activator (26 mmol/L), Mg^2+^ (10 mmol/L), diadenosine pentaphosphate (0.01 mmol/L), hexokinase (>4 kU/L), and glucose-6-phosate dehydrogenase (>2.8 kU/L). Prior to the placement of the reagents on board the instrument, the entire contents of R1-2 was transferred to the entire contents of R1-1 and mixed by gentle inversion. The second reagent bottle (R2) was ready to use from the kit and was placed directly on the instrument.

#### Glucose

The concentration of glucose was determined on an automatic clinical analyser (Olympus AU400 Clinical Analyser, Tokyo, Japan) using the reagents supplied by Olympus (catalogue number OSR6121). The composition of the reagents (at final concentration of reactive ingredients) required for this test were; piperazine-*N*,*N*′-bis (ethanesulfonic acid (PIPES) buffer (pH 7.6) (24 mmol/L), ATP (≥2 mmol/L), NAD^+^ (≥1.32 mmol/L), Mg^2+^ (2.37 mmol/L), hexokinase (≥0.59 mmol/L), G6P-DH (≥0.59 mmol/L), and required no preparation prior to placement on the instrument.

#### Non-esterified fatty acids

The concentration of non-esterified fatty acids (NEFA) was determined on an automatic clinical analyser (Olympus AU400 Clinical Analyser, Tokyo, Japan) using the reagents supplied by Randox Laboratories (catalogue number FA115) (Randox Labs Ltd., Ardmore, Diamond Rd., Crumlin BT29 4QY, Co. Antrim, Ireland). The kit comprised of five reagents. The buffer (R1a) and enzyme diluent (R2a) were ready to use from the kit. The enzyme/coenzymes (R1b) were reconstituted with 10 mL of buffer (R1a) and mixed by gently swirling. Maleimide (R2b) was reconstituted with enzyme diluent (R2a) and was inverted several times to ensure that maleimide was completely dissolved. This was then used immediately to reconstitute enzyme reagent (R2c) which was protected from light and placed on board the instrument for analysis. All reagents were kept at 4 °C prior and after preparation.

#### *β*-hydroxy butyrate (βHB)

The concentration of βHB was determined on an automatic clinical analyser (Olympus AU400 Clinical Analyser, Tokyo, Japan) using the reagents supplied by Randox Laboratories (catalogue number RB 1007). The kit comprised of five reagents which were stored at 4 °C prior and after preparation.

#### Urea

The concentration of urea was determined on an automatic clinical analyser (Olympus AU400 Clinical Analyser, Tokyo, Japan) using the reagents supplied by Olympus (catalogue number OSR6134). All reagents were stored at 4 °C. The composition of the reagents (at final concentration of reactive ingredients) required for this test were; Tris buffer (100 mmol/L), NADH (≥0.26 mmol/L), tetra-sodium disulphate (10 mmol/L), EDTA (2.65 mmol/L), 2-oxoglutarate (≥9.8 mmol/L), Urease (≥17.76 kU/L), ADP (≥2.6 mmol/L), glutamate dehydrogenase (GLDH) (≥0.16 kU/L), and required no preparation prior to placement on board the instrument.

#### Haematology

Haematology profiles were determined using 6 mL of K_3_ EDTA whole blood using an automated haematolgy analyser (Celltac MEK-6108K; Nihon-Kohdon, Tokyo, Japan) and reagents supplied by Celltac (Alpha Technologies, Dublin, Ireland).

#### Haptoglobin

A colorimetric assay (Phase™ Range Haptoglobin Assay, Tridelta Development Ltd., Co. Kildare, Ireland) was used to determine plasma haptoglobin concentration. The haptoglobin kit contained all necessary reagents and the method was based on that of Eckersall et al. [27].

#### Interferon (IFN)-γ

The lymphocyte production of IFN-γ was determined following stimulation in vitro with either phosphate buffered saline alone, keyhole limpet haemocyanin (KLH; 20 µg/1.5 mL blood), or concanavalin A (Con A; 20 µg/1.5 mL blood) in whole blood culture, and subsequently, the IFN-γ concentration in the harvested plasma samples was measured using an ELISA procedure [28].

### Data analysis

The statistical analyses were performed using SAS software version 9.3 (SAS Inst. Inc., Cary, NC, USA). Growth, slaughter performance, dirt scores, hoof lesions scores and physiological data were tested for normality using PROC UNIVARIATE and the Shapiro–Wilk test. The statistical model included floor type, their interactions and block. If the interaction term was not statistically significant (*P* > 0.05), it was subsequently excluded from the final model. The MIXED procedure of SAS (9.3) was used to examine the effect of slats, mats and wood-chip. The experimental unit was animal for all variables except for intakes and behaviour for which pen was the experimental unit. Dirt scores had multiple observations and were analysed using repeated measures ANOVA (MIXED procedure of SAS 9.3) with terms for slat, mat, wood-chip, time and their interactions included in the model. Differences were determined by F-tests using Type III sums of squares. The PDIFF option was applied as appropriate to evaluate pair-wise comparisons between the group means and the associated *P* values were derived. In the full model, treatment, block, day, time and the interaction between treatment and day and treatment and time were included as fixed explanatory variables; animal as random variable, and day as repeated variable. For blood variables, baseline values (mean of values for day 1) were included as a covariate. For the behavioural data, the number of animals performing each activity every 10 min was recorded and the data was transformed in percentages of occurrences of each behaviour. Model effects were considered statistically significant when Type I error rate was less than 5 %. Non-transformed data are presented as least square means (Lsmeans) and SEM to facilitate interpretation of results.

## Results

### Animal diet

The chemical composition and nutritive value of the TMR offered to animals was crude protein 145.3 (SE) 1.5 g/kg dry matter (DM), acid detergent fibre (ADF) 229.3 (3.9) g/kg DM, ash 79.6 (5.3) g/kg DM, nitrogen 23.4 (SE) 0.3 g/kg DM, pH 4.1 (SE) 0.1, DM 316.9 (SE) 6.8 g/kg and dry matter digestibility (DMD) 773.2 (3.9) g/kg DM.

### Animal performance

There was no effect of treatment on performance characteristics of finishing steers (Table 1).

**Table 1:** Effect of floor type on performance characteristics of finishing beef steers

	Slats	Mat 1	Mat 2	Wood-chip
Total DM intake (kg DM/day)	9.9 ± 0.30	9.9 ± 0.06	9.8 ± 0.17	10.1 ± 0.09
Initial live weight (kg)	502.0 ± 7.76	504.4 ± 8.76	502.4 ± 9.32	502.0 ± 8.83
Slaughter weight (kg)	673.6 ± 7.79	672.7 ± 10.43	666.8 ± 10.45	677.35 ± 10.94
Live weight gain (kg/day)	1.16 ± 0.05	1.14 ± 0.05	1.11 ± 0.04	1.18 ± 0.05
Carcass weight (kg)	350.9 ± 4.40	353.9 ± 4.43	348.5 ± 5.43	346.3 ± 5.98
Estimated carcass gain (g/day)^a^	675.5 ± 19.1	687.5 ± 16.7	657.4 ± 19.8	644.5 ± 27.0
Dressing percent (%)	52.1 ± 0.43	52.8 ± 0.69	52.3 ± 0.39	51.1 ± 0.34
Carcass conformation score^b^	2.64 ± 0.12	2.60 ± 0.12	2.78 ± 0.12	2.56 ± 0.12
Carcass fat score^c^	2.93 ± 0.11	2.98 ± 0.11	2.93 ± 0.11	2.57 ± 0.12
Kidney channel fat (kg)	11.38 ± 0.37	12.3 ± 0.54	12.4 ± 0.39	11.01 ± 0.60

The values are expressed as Lsmeans (±SEM)

^a^Assuming an initial dressing percentage of 520 g/kg

^b^Scale EUROP with P = 1 and E = 5

^c^Fat score scale 1 (leanest) to 5 (fattest)

### Animal behaviour

Overall (period 1–5 combined), the percentage of animals lying at any one time, was greater (*P* < 0.05) in the animals housed on mats 1 and 2 compared with the animals housed on slats and on the wood-chip treatment (Table 2). The percentage of animals eating at any one time, was greater (*P* < 0.05) in the animals housed on the wood-chip treatment compared with animals housed on slats, mat 1 and mat 2. There was no difference (*P* > 0.05) in drinking behavior among treatments. The percentage of animals head butting at any one time, was greater (*P* ≤ 0.05) on mat 1 and wood-chip compared with animals housed on slats. There was no significant difference between mat 2 and the other treatments. The percentage of animals showing mounting behavior at any one time was greater (*P* < 0.05) for mat 2 compared with wood-chip and mat 1. The slat treatment showed a greater percentage (*P* < 0.05) of mounting behaviour compared with mat 1. The percentage of animals displaying grooming behaviour, such as licking, at any one time, was greater (*P* < 0.05) on mat 2 compared with mat 1 and wood-chip treatments.

**Table 2:** Effect of floor type on the percentage occurrences of lying, eating, drinking, head butting, mounting, licking and allo-grooming over five 3-day sampling periods

Behaviour	Slats	Mat 1	Mat 2	Wood-chip
Lying	49.6^a^ ± 0.38	51.3^b^ ± 0.39	51.0^b^ ± 0.38	49.5^a^ ± 0.39
Eating	10.3^b^ ± 0.19	10.3^b^ ± 0.20	10.5^b^ ± 0.20	11.2^a^ ± 0.20
Drinking	0.31 ± 0.02	0.30 ± 0.03	0.32 ± 0.03	0.32 ± 0.03
Head butting	0.26^a^ ± 0.02	0.36^b^ ± 0.02	0.32^a,b^ ± 0.02	0.34^b^ ± 0.02
Mounting	0.10^a,c^ ± 0.01	0.05^b^ ± 0.01	0.12^c^ ± 0.01	0.07^a,b^ ± 0.01
Licking another animal (allogrooming)	2.12^a,b^ ± 0.06	1.98^a^ ± 0.06	2.25^b^ ± 0.06	2.0^a^ ± 0.06
Grooming (self grooming)	2.80^a,c^ ± 0.06	2.57^b^ ± 0.07	2.85^c^ ± 0.07	2.37^d^ ± 0.07

The values are expressed as Lsmeans (±SEM) over the five sampling periods

5 three-day sampling periods (1–5) (period 1, days 2, 3, 4; period 2, days 25, 28, 29; period 3, days 42, 43, 44; period 4, days 56, 57, 58 and period 5, days 129, 130 and 133) for steers housed on bare concrete slats

^a,b,c,d^Within a row, means having different superscript letters indicate significant differences (*P* < 0.05)

### Hoof lesions

The total number of lesions on the hooves of the animals was greater (*P* < 0.05) on mat 1, mat 2 and wood-chip treatments compared with the animals on concrete slats (Table 3).

**Table 3:** Effect of floor type on hoof condition of finishing beef steers

	Slats	Mat 1	Mat 2	Wood-chip
Number of hoof Lesions	22.3^a^ ± 2.28	35.5^b^ ± 2.21	40.9^c^ ± 2.21	34.1^b^ ± 2.21
Number of Erosions	11.8^a^ ± 0.82	16.8^b^ ± 0.82	16.1^b^ ± 0.81	10.3^a^ ± 0.85
Number of overgrowths	5.3^a^ ± 0.64	12.5^b^ ± 0.65	13.4^b^ ± 0.64	10.7 ^a^ ± 0.64
Number of white line lesions	22.5^a^ ± 2.29	35.3^b,c^ ± 2.19	41.0^b^ ± 2.22	33.8^c^ ± 2.22

The values are expressed as Lsmeans (±SEM)

^a,b,c^Within a row, means having different superscript letters indicate significant differences (*P* < 0.05)

The level of erosion was greater (*P* < 0.05) in animals on mat 1 and mat 2 compared with those on slats and wood-chip (Table 3). The quantity of overgrowth was greater (*P* < 0.05) on mat 1, mat 2 and wood-chip compared with those animals on slats (Table 3). The mat 1 treatment showed greater overgrowth (*P* < 0.05) compared with animals on the wood-chip treatment. Mat 1, mat 2 and wood-chip treatments had greater white line dominance (*P* < 0.05) compared with slats. Mat 2 treatment had greater white line dominance (*P* < 0.05) compared with the wood-chip treatment (Table 3).

### Dirt scores

There was no difference (*P* > 0.05) in dirt scores between treatment groups at the start of the study (Table 4). On day 23, animals housed on wood-chip had greater (*P* < 0.05) dirt scores than those on slats, mat 1 and mat 2. However, animals housed on mat 2 had lower dirt scores (*P* < 0.05) compared with the other treatments. Animals housed on the wood-chip had greater (*P* < 0.05) dirt scores on day 45, 65, 86, 107, 128 and 148 compared with the other treatments.

**Table 4:** Effect of floor type on dirt scores of finishing beef steers

	Slats	Mat 1	Mat 2	Wood-chip
Day 0	42.0^x^ ± 1.13	43.7^x^ ± 1.15	43.2^x,y^ ± 1.13	43.4^x^ ± 1.13
Day 23	49.8^x^ ± 1.39	49.5^a,x^ ± 1.41	45.5^b,x,y^ ± 1.39	58.3^c,x^ ± 1.39
Day 45	41.2^a,x^ ± 1.42	42.7^a,x^ ± 1.44	40.9^a,x,y^ ± 1.42	59.3^b,x^ ± 1.44
Day 65	33.9^a,y^ ± 1.30	33.0^a,y^ ± 1.32	35.1^a,y^ ± 1.30	54.3^b,x^ ± 1.30
Day 86	26.1^a,y^ ± 1.26	26.3^a,y^ ± 1.28	26.6^a,y^ ± 1.26	41.1^b,x^ ± 1.26
Day 107	20.4^a,y^ ± 0.95	20.3^a,y^ ± 0.96	21.6^a,y^ ± 0.95	33.1^b,y^ ± 0.95
Day 128	21.3^a,y^ ± 1.20	20.2^a,y^ ± 1.21	20.5^a,y^ ± 1.20	59.6^b,y^ ± 1.20
Day 148	20.9^a,y^ ± 0.94	20.2^a,y^ ± 0.96	20.8^a,y^ ± 0.94	46.4^b,x^ ± 0.94

Values are expressed as Lsmeans dirt score (±SEM)

The left side of each animal was diagrammatically divided into 16 segments and each body segment was assigned a score between 1 (very clean) and 5 (very dirty). All scores were summed to obtain a total score for each animal, between 1 and 80, which equated to the sum of the scores for each of the 16 body segments

^a,b,c^Within a row, means having different superscript letters indicate significant differences (*P* < 0.05)

^x,y^Within a column, means having different superscript letters indicate significant differences (*P* < 0.05)

### Physiological variables

There was no floor type × sampling time interaction (*P* > 0.05) or effect of floor type (*P* > 0.05) among treatments on day 23, 45, 86, 107, 128 and 148 for plasma albumin, globulin, protein, creatine kinase, glucose, *β*-hydroxy butyrate, fibrinogen, and haptoglobin concentrations, white blood cell number and IFN-γ production (data not shown). There was a treatment × interaction (*P* = 0.005) for plasma NEFA concentrations (Table 5) and an effect of time (*P* = 0.0001). There were no differences (*P* > 0.05) in plasma NEFA concentration between treatments (floor type) (Table 5) during the housing period on days 23 and 45. Plasma NEFA concentrations decreased in animals housed on mat 2, slats and wood-chip on day 65. On days 107, 128 and 148, plasma NEFA concentrations were greater compared with baseline for animals housed on mat 2, concrete slats and the wood-chip treatments.

**Table 5:** Effect of floor type on plasma non-esterified fatty acids concentrations, haematocrit %, red blood cell (RBC) number and plasma haemoglobin concentration

		Day 23	Day 45	Day 65	Day 107	Day 128	Day 148
Non-esterified fatty acids (mmol/L)	Slats	0.12 ± 0.01^a^	0.09 ± 0.01	0.11 ± 0.01^b^	0.17 ± 0.10^b^	0.14 ± 0.01^b^	0.20 ± 0.02^b^
	Mat 1	0.12 ± 0.01	0.08 ± 0.01	0.10 ± 0.01	0.12 ± 0.01	0.12 ± 0.01	0.18 ± 0.02
	Mat 2	0.11 ± 0.01^a^	0.09 ± 0.01	0.10 ± 0.01^b^	0.10 ± 0.01^b^	0.12 ± 0.01^b^	0.16 ± 0.02^b^
	Wood-chip	0.13 ± 0.11^a^	0.10 ± 0.01	0.09 ± 0.01^b^	0.13 ± 0.01^b^	0.15 ± 0.01^b^	0.18 ± 0.02^b^
	Treatment *P* = 0.164; Time *P* = 0.0001; Treatment × time *P* = 0.005						
Haematocrit %	Slats	32.4 ± 0.84^a^	35.5 ± 0.76	36.5 ± 0.80^b^	37.8 ± 0.66^b^	37.8 ± 0.74	36.2 ± 0.71
	Mat 1	33.1 ± 0.83^a^	35.0 ± 0.78	35.7 ± 0.80^b^	37.9 ± 0.67^b^	37.8 ± 0.74^b^	36.7 ± 0.73^b^
	Mat 2	34.1 ± 0.83^a^	33.2 ± 0.75	36.6 ± 0.80^b^	37.6 ± 0.65	37.6 ± 0.74^b^	37.1 ± 0.71
	Wood-chip	34.2 ± 0.83	33.7 ± 0.76	35.7 ± 0.80	35.4 ± 0.65	37.2 ± 0.74	35.2 ± 0.70
	Treatment *P* = 0.569; Time *P* = 0.0001; Treatment × time *P* = 0.006						
Red Blood cell (×10^6^ cells/μL)	Slats	8.0 ± 0.10^a^	8.3 ± 0.11^b^	8.4 ± 0.10^b^	8.6 ± 0.10^b^	8.8 ± 0.11^b^	8.8 ± 0.11^b^
	Mat 1	8.0 ± 0.10^a^	8.2 ± 0.12	8.3 ± 0.10^b^	8.8 ± 0.11^b^	8.7 ± 0.11^b^	8.6 ± 0.11^b^
	Mat 2	8.0 ± 0.10^a^	8.0 ± 0.11	8.3 ± 0.10	8.7 ± 0.10^b^	8.7 ± 0.11^b^	8.7 ± 0.11^b^
	Wood-chip	8.2 ± 0.10^a^	8.0 ± 0.12	8.1 ± 0.10	8.5 ± 0.10	8.6 ± 0.11^b^	8.5 ± 0.11
	Treatment *P* = 0.42; Time *P* = 0.0001; Treatment × time *P* = 0.008						
Haemoglobin (g/dL)	Slats	10.8 ± 0.14	11.3 ± 0.16	13.8 ± 0.16^b^	14.0 ± 0.15^b^	13.6 ± 0.15^b^	13.3 ± 0 .17^b^
	Mat 1	10.9 ± 0.14	11.2 ± 0.16	13.9 ± 0.16^b^	14.0 ± 0.15^b^	13.6 ± 0.15^b^	13.3 ± 0.17^b^
	Mat 2	11.0 ± 0.14	10.7 ± 0.16	13.9 ± 0.16^b^	13.9 ± 0.15^b^	13.6 ± 0.15^b^	13.5 ± 0.17^b^
	Wood-chip	11.0 ± 0.14	10.7 ± 0.16	13.5 ± 0.16^b^	13.3 ± 0.15^b^	13.3 ± 0.15^b^	12.8 ± 0.17^b^
	Treatment *P* = 0.09; Time *P* = 0.0001; Treatment × time *P* = 0.0006						

Pre-housing blood measurements were taken day 0. Values are expressed as Lsmeans (±SEM) with *P* values

^a,b^Within a row, means having different superscript letters indicating significant differences (*P* < 0.05)

There was a treatment × interaction (*P* = 0.006) and an effect of time for haematocrit (Table 5). There was no difference (*P* > 0.05) in haematocrit between treatments (floor type) (Table 5) on day 45. On day 65, haematocrit increased in animals on mat 1, mat 2 and slats compared with baseline, while on day 107 values were increased in animals on mat 1 and on slats. On day 128, haematocrit was increased in animals on mat 1 and mat 2 compared to baseline, and by day 148 only the mat 1 treatment had greater values than baseline.

There was an effect of treatment (*P* < 0.001), no effect of time (*P* = 0.09) time, and no treatment × time interaction (*P* = 0.07) for lymphocyte number. There was an effect of treatment (*P* < 0.004), an effect of time (*P* = 0.0003) time, and no treatment × time interaction (P = 0.06) for neutrophil number. Neutrophil number [Lsmeans (SEM)] decreased in animals on mat 2 on day 23 [26.4 (0.051)], day 45 [22.7 (0.99)] and day 148 [23.7 (0.64)] compared with baseline values.

There was no effect of treatment (*P* = 0.42), an effect of time (*P* = 0.0001) time, and a treatment × time interaction (*P* = 0.008) for red blood cell (RBC) number (Table 5). On days, 65, 107, 128 and 148, RBC number was increased in animals on mat 1 compared to baseline; on days 107, 128 and 148, RBC number was increased in animals on mat 2 compared to baseline; animals on slats had increased RBC number from day 45 to day 148 compared to baseline, whereas, animals on the wood-chip treatment had increased RBC number on day 128 only compared with baseline.

There was no effect of treatment (*P* = 0.09), an effect of time (*P* = 0.0001) time, and a treatment × time interaction (*P* = 0.0006) for plasma haemoglobin concentrations (Table 5). Plasma haemoglobin concentrations were increased in all treatments compared with baseline on days 65, 107, 128 and 148 of the study.

There was no effect of treatment (*P* = 0.195), an effect of time (*P* = 0.004) time, and a treatment × time interaction (*P* = 0.0003) for plasma cortisol concentrations. Plasma cortisol concentrations [Lsmeans (SEM ng/mL)] were increased in animals on mat 1 on days 107 [28.2 (2.34)], 128 [29.4 (2.43)] and 148 [29.9 (2.5)] compared to baseline values (18.4 ± 1.67). On day 107, plasma cortisol concentrations were increased in animals on mat 2 [22.4 (2.3)] compared with baseline [16.3 (1.63)]. Animals on the wood-chip treatment had greater (*P* < 0.05) cortisol concentrations on day 107 [23.0 (2.28)], day 128 (24.9 (2.4)) and day 148 [19.8 (2.41)] compared to day 0 {baseline [13.2 (1.63)]}. There was no change in plasma cortisol concentrations over time in animals housed on slats. There was no effect (*P* > 0.05) of treatment, of time, or treatment × time interactions for either phytohaemagglutinin A (PHA) induced or concanavalin A (CON A) induced IFN-γ production (data not shown).

### Environmental conditions

The mean daily air temperature (±SEM) recorded in the shed facility was 10.5 °C (0.02) (Min 1.5 Max 23.5) and 7.2 °C (0.04) (Min −6.5; Max 26.7) for the environmental (outside) temperature.

## Discussion

Fully slatted concrete floors are in use throughout Europe for housing beef cattle during the winter [29]. In Ireland, due to the seasonality of grass growth the production systems in use for rearing and finishing beef cattle usually consist of a grazing season of 8 months followed by a winter housing period of 4–5 months duration [30]. Concrete slatted floors are the predominant winter housing system in use [31]. In the present study, the housing comparison, concrete slats versus other underfoot conditions (mat 1, mat 2 and wood-chips) was chosen because prolonged standing on concrete is recognized as one of the major risk factors for lameness [32]. The findings of the present study are in agreement with Lowe et al. [33] where no significant effect of floor type on production parameters such as live-weight, carcass gains and carcass composition was reported. Similarly, in cattle housed on a range of floor types, including fully slatted floors covered with perforated rubber mats, fully slatted floors, rubber strips secured directly onto slats, and straw bedding, Kirkland and Steen [34] found no difference in feed intake or growth rate. By contrast, Irps [2] reported a greater live weight gain in bulls housed on floors with strips of rubber compared with those housed on fully slatted flooring.

Recently, concerns have been expressed regarding the cleanliness and lameness, of beef cattle housed on concrete slatted floors [35–38]. Covering concrete slats with rubber mats is one alternative to exposed concrete slats that has been proposed. However, studies examining the effect of concrete slats and rubber mats on the welfare of beef cattle are limited [39].

Contrasting results have been obtained with regard to the cleanliness of animals housed in slatted units with or without rubber coatings [4, 40]. Hultgren and Bergsten [4] reported that cattle housed on slats alone showed a greater risk of getting dirty when compared with rubber slatted flooring. However, it is suggested that the cleanliness of animals within a housing system depends on whether or not there are clean areas available for lying [25]. Lowe et al. [33] reported no difference in dirtiness between cattle on slats, rubber strips or straw, whereas cattle on rubber mats were significantly dirtier than those on rubber strips or straw. Interestingly, in the present study, steers housed on the wood-chip treatment had greater dirt scores compared with the other three treatments and may be related to wetter underfoot conditions.

Most of the published research to date on the effect of floor type on hoof health of cattle has been conducted mainly using dairy cattle [40–44]. In terms of hoof health, the present study showed a greater number of hoof lesions due to covering concrete slats with rubber mats. Graunke et al. [45] reported that bulls on concrete slats had a greater number hoof lesions than bulls housed on rubber mats. Schulze Westerath et al. [46] reported a lower incidence of lesions and lower occurrence of swellings of the joints in bulls housed on rubber coated compared to bare concrete slats. While no slipping was observed in the present study for bulls on concrete slats, Smits et al. [47] reported less slipping for bulls on rubber-coated compared with conventional slats. In the present study, steers housed on slats showed least erosion of the hoof, least white line dominance, least overgrowth and the least degree of lesions when compared with steers housed on slats with mats. Similarly, Schlichting [48] reported that hoof growth and wear was greatest on concrete, intermediate on the rubber-coated slats, and least on fully-straw bedded sheds for calves. Other studies have shown that general necrotic lesions were more common in straw-bedded cattle when compared with animals housed in slatted housing systems suggesting that wet conditions soften the hoof horn and weaken the skin barrier therefore increasing the chances of bacteria invading the hoof tissue. Kirkland and Steen [34] reported no incidence of clinical lameness in any of the four treatments in their study (i.e. slats, slats with mats, slats with strips or straw). Similarly, in our study, no signs of lameness were detected. In contrast to our study, Hannan and Murphy [1] reported the incidence of lameness was 4.75 % among steers housed on slatted floors compared with 2.43 % for steers in straw yards.

In the present study, the percentage of animals lying at any one time was greater in the animals housed on rubber coated slats compared with the animals housed on slats and on the wood-chip treatment. Kirkland and Steen [34] reported that cattle on straw stood up and lay down more frequently than cattle housed on slats. In the present study, the percentage of animals eating at any one time was greater in the animals housed on the wood-chip treatment compared with animals housed on slats, or either rubber coated slat. Mounting activity has been shown to be greater when underfoot conditions allow animals to move more freely [49, 50]. In the present study, mounting activity was greater on slats with rubber mats fitted. It is likely that the rubber mats improved comfort and steers’ confidence to stand and move.

In our study the results indicate that there was no major effect of floor type on blood metabolite concentrations, suggesting a metabolic homeostasis across treatments [51–54]. Plasma albumin, globulin, glucose, *β*-hydroxy butyrate concentrations and creatine kinase activity, were, for the most part, not different between animals housed on slats and animals housed on the other treatments, suggesting that animals were not undergoing metabolic stress.

White blood cell (WBC) percentages were similar among treatments. Changes in the composition of haematological profiles may reflect the physiological and pathophysiological response of animals to stress [53, 54]. Studies measuring stress related immune blood variables in cattle has primarily focused on husbandry practices, for example, weaning of beef calves [55–57], castration of bulls [28, 58], animal transport [59–62] and restricted space allowance during housing [39, 63–65]. In the present study, neutrophil number decreased in animals on mat 2 on days 23, 45 and 148 compared with baseline values. Neutrophils are the first line of defense and play a major role in removing invading bacteria. A reduction in IFN-γ poduction in response to in vitro stimulation with a mitogen in conjunction with a reduction in lymphocyte and an increase in neutrophil percentages is indicative of reduced immune function. However, in this study there was no difference in Con-A or PHA induced IFN-γ production among treatments. Therefore the degree of immunosuppression attributed to the increase in neutrophil percentages is not supported by changes in immune responsiveness. Cortisol is an important indicator of stress in animals [39, 63–65] however, in the present study, plasma cortisol concentrations were similar for all treatments. An increase in plasma cortisol concentration together with the suppression of immune function through the suppression of lymphocyte blastogenesis and IFN-γ production is the key defining feature of the stress response in cattle. Cortisol concentrations were, in general, similar across all treatments throughout the present study.

In conclusion, under the conditions of the present study, there was no evidence to suggest a performance or welfare benefit in terms of physiology, dirt scores, behavior and hoof lesions to animals, whether they were housed on a particular mat type (mat 1 or mat 2) or on wood-chip, compared with animals housed on bare concrete slats. Further studies on underfoot comfort for finishing cattle are warranted together with performing a meta-analysis of published housing data.

## Abbreviations

ADF: acid detergent fibre
bHB: beta hydroxy butyrate
DM: dry matter
DMD: dry matter digestibility
CCTV: close circuit television
Con-A: concanavalin-A
IFN-γ: interferon-γ
PHA: phytohaemagglutin A
kg: kilogram
NEFA: non-esterified fatty acids
RBC: red blood cell
SE: standard error of the mean
TMR: total mixed ration
WBC: white blood cell
